# Reimagining land system science in seascapes

**DOI:** 10.1007/s10980-026-02429-5

**Published:** 2026-08-05

**Authors:** Elizabeth O. Ruff, Matthew Clark, Lucas Alencar, Katie Devenish, Philip A. Loring, Sandy Nofyanza, Johan A. Oldekop, Mary A. Young, Joshua E. Cinner

**Affiliations:** 1 Thriving Oceans Research Hub, School of Geosciences, University of Sydney, Camperdown, NSW Australia; 2 Global Development Institute, University of Manchester, Manchester, United Kingdom; 3 Global Science Team, The Nature Conservancy, Arlington, Virginia United States; 4 Deakin Marine Science Centre, School of Life and Environmental Sciences, Deakin University, Warrnambool, Victoria Australia

**Keywords:** Seascape ecology, Sea system science, Social-ecological systems, Seascape use, Land-sea analogs, Ocean ontologies

## Abstract

**Context:**

Terrestrial concepts often guide the science and management of marine systems, as exhibited by the influence of landscape ecology on seascape ecology. However, the theoretical assumptions that inform these concepts have not been sufficiently interrogated within the distinct social-ecological realities of seascapes.

**Objective:**

We call for a ‘sea system science’ that navigates the human dimensions of seascape change to advance the study and management of ocean spaces.

**Methods:**

We evaluate two terrestrial concepts (intensification and expansion) that underpin common approaches to marine management. We establish key dimensions of theoretical (mis)alignment across agriculture, forestry, hunting, marine aquaculture, and capture fisheries.

**Results:**

Seascape uses are substantively different from their landscape counterparts, though there is limited alignment between agriculture and marine aquaculture and between hunting and capture fisheries. We outline opportunities for sea system science to both interrogate terrestrial management concepts and develop an alternative management ontology tailored to seascapes.

**Conclusions:**

A sea system science that engages with the human dimensions of seascape change and co-evolves with seascape ecology offers a pathway toward management approaches that are grounded in the social-ecological realities of marine spaces. Without closer attention to these dimensions, seascape ecology risks implementing terrestrial management practices that are procedurally feasible but ecologically and socioeconomically ineffective.

## Introduction

Landscape ecology is an interdisciplinary field that focuses on the patterns and processes that shape terrestrial ecosystems across space and through time. Anchored in biological and ecological ontologies, landscape ecology has made critical contributions to our collective knowledge of terrestrial systems at varying scales (Turner 2005). These include key concepts related to habitat fragmentation (Fischer & Lindenmayer 2007; Wu 2013), ecosystem service delivery (Duarte et al. 2018; Iverson et al. 2014), and population dynamics (Wiens 1997; Wiens et al. 1993). As landscape ecology describes biophysical processes in terrestrial spaces, the parallel and complementary field of land system science grapples with the human dimensions of change across landscapes. Drawing on the social, environmental, and geographic sciences, as well as complex systems theory, land system science studies how human activities both shape and are shaped by terrestrial ecological patterns (Iwamura et al. 2018; Turner et al. 2007; Verburg 2006). For example, land system science has assessed how different governance regimes influence forest cover and poverty outcomes (e.g., Den Braber et al. 2024), linked farmers’ adaptive capacity to forest biomass (e.g., Wells et al. 2020), and demonstrated the potential for policy leakages to displace deforestation (e.g., Meyfroidt & Lambin 2009). Though landscape ecology and land system science differ in the questions they pose and the conceptual foundations they employ, together they provide a basis for explaining, theorizing, and managing a range of phenomena across terrestrial social-ecological systems (Ban et al. 2013; Iwamura et al. 2018; Stephanson & Mascia 2014).

Landscape ecology is not only restricted to terrestrial spaces but also considerably underpins the ways we study the sea, to the extent that its ocean counterpart—seascape ecology—is often simply described as “the application of landscape ecology concepts to the marine environment” (Pittman 2017). Specifically, seascape ecology seeks to understand the ecological outcomes of spatial and temporal processes across marine spaces (i.e., seascapes) (Pittman et al. 2021; Swanborn et al. 2022). Although the field has evolved from its terrestrial foundations (Wedding et al. 2025), seascape ecology continues to leverage many of the concepts, methodologies, and tools employed by landscape ecology. For instance, seascape ecologists have applied principles of landscape patch dynamics to evaluate spatial complexity in seagrass meadows (Van Der Heide et al. 2012), assess the importance of habitat composition on tropical fish assemblages (Moustaka et al. 2024), and question whether coral reef fragmentation is exacerbated by Crown-of-thorns starfish (Cumming et al. 2025). Like landscape ecology, seascape ecology is increasingly viewed as a solution-focused science that informs the development of marine management and conservation strategies (Dubois et al. 2016; Pittman 2017; Wedding et al. 2025; Weeks 2017).

Unlike landscape ecology, seascape ecology lacks a co-evolving, concomitant field that engages with the intertwining socioeconomic processes and ecological dynamics of marine spaces. Although a substantial body of marine social-ecological research has developed across fisheries (e.g., Basurto et al. 2013; Stephenson et al. 2018), aquaculture (e.g., Johnson et al. 2019; Partelow et al. 2025), and management (e.g., Bigagli 2016; Partelow et al. 2019) contexts, this work has not coalesced into generalized middle-range theories of seascape change—analogous to land system science (Meyfroidt et al. 2018)—leaving a structural gap in how we study and manage seascapes. By default, this gap has been filled by concepts and tools designed to understand and manage land systems that are predicated on land system theories and their requisite assumptions. For example, prioritization models developed for terrestrial spaces are routinely used to design marine parks (e.g., Abarca et al., 2022; Galparsoro & Borja, 2021; Smith et al., 2009), inform marine spatial plans (e.g., Guerry et al. 2012; Kirkman et al. 2019), and identify development areas for marine aquaculture (e.g., Henriques et al. 2017). These concepts and tools have been ‘adapted’ to seascapes without a thorough interrogation of their suitability to marine contexts beyond technical feasibility, and the solution logics produced (underpinned by land-based assumptions) are potentially misaligned with the social, ecological, and material dynamics of seascapes.

As such, a distinct ‘sea system science’—one that engages with the human dimensions and socioeconomic drivers of ecological patterns and changes in seascapes—is needed. Conceived as complementary to, rather than a replacement of, marine social-ecological systems research, sea system science seeks to synthesize insights from marine social science, marine social-ecological systems, ocean governance, and ecological research into generalized theories of change that inform seascape-centered research and management strategies. This proposed research arena should identify recurrent cause-and-effect relationships and interactions between social and ecological processes across spatial scales and the role of key contextual factors in moderating processes (Meyfroidt et al. 2018; Muthukrishna & Henrich 2019). Building this distinct area of research, with its theories and empirical evidence, is essential for not only generating and refining our understanding of seascape change but also unifying existing streams of social-ecological research across terrestrial and marine systems.

Existing social-ecological systems literature has advanced our understanding of human-nature interactions within marine systems. Examples include developing frameworks for the inclusion of social metrics into ecological analyses (e.g., Lindkvist et al. 2022), incorporating fisher decision-making into fisheries models (e.g., Lade et al. 2015; Wijermans et al. 2020), understanding the impacts of marine aquaculture on community well-being (e.g., D’Anna & Murray 2015), and testing how human–environment theories hold up empirically in marine spaces (e.g., Cinner et al. 2022). Aswani (2019) even called for the development of a specific coastal human ecology corpus to elucidate human interactions with seascapes at the local scale, with Bennett (2019) similarly emphasizing the role for marine social science in creating sustainable seas. However, for the most part, this literature has explored marine social-ecological relationships through direct linkages (e.g., fish and fishers), lacking a relational spatial focus on the indirect processes (e.g., power, politics, market forces, spillovers) that drive human activities and ecological change as they overlap across seascapes (Crawford et al. 2022).

Here, we make the case for advancing a deliberate sea system science agenda by evaluating how two key land system processes—intensification and expansion of productive use—translate to marine systems. Mechanistic theories of improving per-unit land productivity (intensification) and converting land for human use (expansion) underpin both the interpretation of past change and the projection of future change in terrestrial systems (Meyfroidt et al. 2018). In making the case for a sea system science, we focus on these two processes as they 1) underpin some of the most widely deployed marine management and conservation strategies, including marine spatial planning and marine protected areas, and 2) provide an accessible heuristic for demonstrating the fundamental tensions between land- and seascapes. However, we note that our approach to interrogating intensification and expansion can be replicated across other terrestrial concepts and processes such as displacement and telecoupling.

Through the lens of five central seascape (fishing and aquaculture) and landscape (agriculture, forestry, and hunting) uses, we identify if and how the mechanisms of intensification and expansion (mis)align across marine and terrestrial realms. This exercise allows us to reveal spaces of knowledge that require ocean-specific ontologies and reflect on the implications of our findings in the context of conservation practice and the potential for new seascape-centered theoretical development.

## Building a sea system science

Building a sea system science requires defining the spatial bounds within which we are considering human uses of the ocean. We acknowledge that terrestrial and marine spaces are inexorably linked across ecological (e.g., Fang et al. 2018; Sandin et al. 2022) and human (e.g., Diver et al. 2019; Gladstone-Gallagher et al. 2022) dimensions and that management strategies need to account for these connections (Delevaux et al. 2018; Tulloch et al. 2021). However, a legacy of treating the sea as an extension of the land (e.g., as sites of expanding urbanization; Couling 2018) or as a blank space between landscapes (e.g., Anderson & Peters 2014), as opposed to its own distinct social-ecological realm, has constrained how we study and manage marine spaces (Crawford et al. 2022). Marine social-ecological scholarship has increasingly recognized and pushed back on this legacy (e.g., Burns 2023; Fairbanks et al. 2018; Havice & Zalik 2018; Steinberg & Peters 2015), and sea system science seeks to consolidate and advance these critiques through seascape-centered theories of change.

Here, we define the ‘sea’ as a socio-natural space that is consistently covered by salt water (inclusive of intertidal areas). Within this scope, we constrain our ‘sea system’ to ocean spaces within 20 km of the coastline, which is where most human activities and their corresponding influences on marine ecosystems occur (Halpern et al. 2025). This boundary excludes the high seas, particularly areas beyond national jurisdictions, as these spaces have their own distinct biophysical and social characteristics that differently shape their social-ecological structures and warrant their own ontological consideration (Lidström et al. 2026). This justification also extends to our exclusion of transition zones such as estuaries, where riverine dynamics and resource accessibility create distinct social-ecological systems (Taylor, and Suthers, 2021), as well as coastal floodplains, mangroves, and other swamp forests, which also have been included in existing land system science research (e.g., Drummond et al. 2015; Hagger et al. 2022). As such, shrimp aquaculture, which has resulted in widespread conversion of mangrove habitats to production, is not included in our conceptualization of marine aquaculture as a seascape use. While these exclusions are supported by literature and facilitate conceptual coherence, we note that ecological and biophysical processes in both the high seas and transitional zones are tightly coupled with those within our defined bounds for sea system science.

There are distinct properties of seascapes that should guide how we (re)think of the deployment of terrestrial concepts in marine spaces. For example, as a physically, socially, and economically fluid environment, the ocean subverts our perceptions of land as an “idealized inert and homogenous space” (Herrera-Racionero et al. 2020) that can be readily parceled into units and categorized based on its primary use (Crawford et al. 2022). While administrative (e.g., Exclusive Economic Zones) and use boundaries (e.g., no-fishing zones) are used to delineate marine spaces, these spatial demarcations are fuzzier and more porous than their terrestrial counterparts. Further, human activities in seascapes are less spatially explicit and often overlapping with multiple activities occurring in the same space at different times (e.g., recreational fishing and marine tourism; shipping and commercial fishing; Voyer et al. 2017). These characteristics make it difficult to clearly demarcate ‘parcels’ of ocean and categorize human uses at sea, further complicating spatially-explicit approaches that are common both to land system science research and to conservation and management strategies applied across land- and seascapes (Kuemmerle 2024). Marine aquaculture deviates to some extent from these fuzzy boundaries as farm perimeters are often physically demarcated by buoys and gear, creating relatively defined spatial footprints. However, this spatial legibility comes with its own distinct seascape tensions as it signals the privatization of the ocean commons and the restriction of access for long-established human uses of the marine space, such as recreational and commercial fishing (Bennett et al. 2015; Fairbanks et al. 2018). Further, while the spatial bounds of marine aquaculture are relatively less fuzzy than other uses of the marine space, they are still porous and, therefore, are subject to the biophysical and socioeconomic fluidity of the sea highlighted above.

Connective processes also operate in fundamentally different ways within land- and seascapes. In terrestrial systems, distance typically serves as a reasonable proxy for both social and ecological connectivity, often weighted by quantifying surface “friction” or travel costs (Andrews et al. 2025; McRae et al. 2008). However, in marine systems, distance is a weak predictor of connectivity (e.g., Thomas et al. 2014). Ocean currents can transport species and nutrients vast distances while simultaneously impeding movement between neighboring areas, and their effects vary widely across organisms, human uses, and geographies. For instance, the productivity of coral reef systems is subsidized by mobile, offshore pelagic species in ways that are still not entirely understood (Skinner et al. 2021). Research concepts on exogenous drivers (e.g., Godar et al. 2015; Meyfroidt 2013) and telecoupling processes in land systems, whereby cross-scale socio-economic and ecological linkages mean local outcomes can be influenced by distal drivers (e.g., Busck-Lumholt et al. 2022; Friis et al. 2016), offer a starting point for navigating the social-ecological implications of the ocean’s distinct connective processes. However, the differences outlined above underscore the likelihood that these terrestrial concepts will need to be reconsidered and adapted for seascapes.

The unique social-ecological attributes of the sea (e.g., fluidity and connectivity) have profound implications for resource governance because they affect the actualization of clear, stable, and enforceable boundaries. As articulated in Elinor Ostrom’s first design principle for cooperative commons management, the visibility and reliability of resource boundaries determine the feasibility of various property regimes (Ostrom 1990). Clearly defined and reliable resource boundaries enable the enforcement of access rights (i.e., who can enter resource areas), which allow for better estimation of sustainable resource use rights (i.e., how much resource can be extracted) (Andrews et al. 2024; Clark et al. 2024a, b). On land, such boundaries are typically well-established: cadastral maps and registries support long-term, legally enforceable property rights that are embedded within nested institutions for zoning, taxation, and planning. In contrast, while access-based tenure systems exist in marine systems (e.g., Muawanah et al. 2021), they generally are much weaker given resource boundaries are fluid and difficult to physically delimit (Acton et al. 2019). As a result, marine governance relies primarily on use rights rather than access rights, making enforcement difficult and contributing to the persistence of de facto open-access conditions (Crawford et al. 2022). These challenges are particularly pronounced in the context of highly mobile resources (e.g., tuna), which are often managed through weakly enforced international law.

Finally, the ocean also challenges land-based processes of valuation. In terrestrial settings, value is mostly determined by the qualities of a unit of land itself—its biophysical characteristics, proximity to markets, and existing infrastructure as well as the availability and value of similarly productive and accessible land (Meyfroidt et al. 2018; Nilsson & Johansson 2013; Plantinga & Miller 2001). However, in the marine context, value is often assigned not to a clearly delineated parcel of the ocean but rather to the resources provided and/or the ecosystem services rendered by the sea (Miller et al. 2017; Samonte-Tan et al. 2007). This dynamic is evidenced by ample peer-reviewed publications, white papers, and government reports calculating the contributions of fisheries, marine aquaculture, and tourism sectors to the blue economy or estimating the financial benefits of coastal protection and carbon sequestration from seascapes. Assigning value to resources themselves and not to the space in which they are produced has important implications for the suitability of land system science concepts that are predicated on land value (e.g., Alonso 1964) as well as conservation and management tools that are underpinned by cost optimization objectives (e.g., Chiarella et al. 2023). For example, the mobility of many marine resources (especially fisheries) confounds land system principles of static, readily bounded parcels that are necessary for identifying fixed spatial tradeoffs. Moreover, the ecosystems that sustain these marine resources (e.g., seagrass beds and kelp forests that function as nursery habitats) are often spatially distant from where harvesting ultimately occurs (Green et al. 2015; Huijbers et al. 2013). Applying land-based valuation principles risks obscuring the contributions of these intermediate habitats and the ecological connectivity pathways linking them to harvest areas. Where land-based valuation directs management investment toward high-value spaces, marine resource valuation does not readily produce such spatial signals, which can leave productive but distal habitats economically invisible within terrestrially-derived optimization frameworks (Colloca et al. 2015).

## (Imperfect) land-sea analogs

We evaluate how the mechanisms of intensification and expansion—and the assumptions they rely on—operate across land and sea systems. We explore these two terrestrial concepts as they underpin common strategies and tools (e.g., systematic conservation planning and marine protected areas) utilized in the management of seascapes. We conduct our assessment within the scope of two dominant anthropogenic uses of seascapes—marine aquaculture and wild-capture fisheries. Aquaculture and fisheries often have been presented as marine equivalents to agriculture and forestry (Gale 1985; Mccay 1981; Napier et al. 2020; Natale et al. 2013), which are two of the most extensive forms of human land use, and both feature prominently in land system theory (Geist & Lambin 2002; Meyfroidt et al. 2018; Sonter et al. 2015). As such, we leverage these landscape uses to ground our assessment both pragmatically and theoretically. Finally, despite the relative exclusion of this activity from land system science literature (Neumann et al. 2022; Sonter et al. 2015), we include hunting in this assessment as it is often positioned as a close analog to wild-capture fisheries (e.g., Mccay 1981; Natale et al. 2013; Renner & Huntington 2014).

Below, we explore the ways in which these land- and seascape uses align and diverge across resource materialities. We consider resource materialities to be the ways that resources are produced and how resources function within the socioecological contexts of their respective ‘scapes’ (Table 1). In this way, our assessment moves beyond the fundamental physical, biological, and ecological differences between land- and seascapes (outlined in Sect. “Building a sea system science”) to evaluate the specific logics that shape intensification and expansion paradigms. The resource materialities presented here are not intended to be an exhaustive list of comparison points between land- and seascape uses but, rather, readily recognizable features of anthropogenic use. We note that our definition of ‘habitat conversion’ in the table below is specific to the transformation of habitat as a necessary input to production (e.g., forest clearing for agriculture) and excludes habitat changes that are an externality or outcome of production. Additionally, while there are likely exceptions to the way we characterize land- and seascape uses below, theory is built on generalizations. In Sect. “Do terrestrial assumptions hold in the sea?”, we discuss how these resource materialities challenge the underlying assumptions of landscape theory when applied to seascapes.

**Table 1:** Resource materialities of land- and seascape uses. Note: our definition of habitat conversion excludes habitat changes that are an externality of production

Resource Materialities	Description
Common property	A resource is fundamentally non-excludable and rivalrous
Habitat conversion	Transformation of natural habitat is a pre-condition for, or input to, resource production
Managed production	Resource is systematically produced (e.g., feed inputs, stocking/harvest schedules, pharmaceutical interventions)
Mobile resource	A resource is not spatially confined and can move naturally across an environment
Primary resource	Resource is harvested or extracted directly from nature and used or consumed with minimal modification to its original state
Resource-based	Production of a resource is dependent upon inputs from other resources (e.g., pastureland for livestock grazing, fishmeal for fed aquaculture)
Stock replacement	A resource can be replaced or regrown through human intervention after it has been harvested or extracted

### Marine aquaculture

**Fig. 1:**
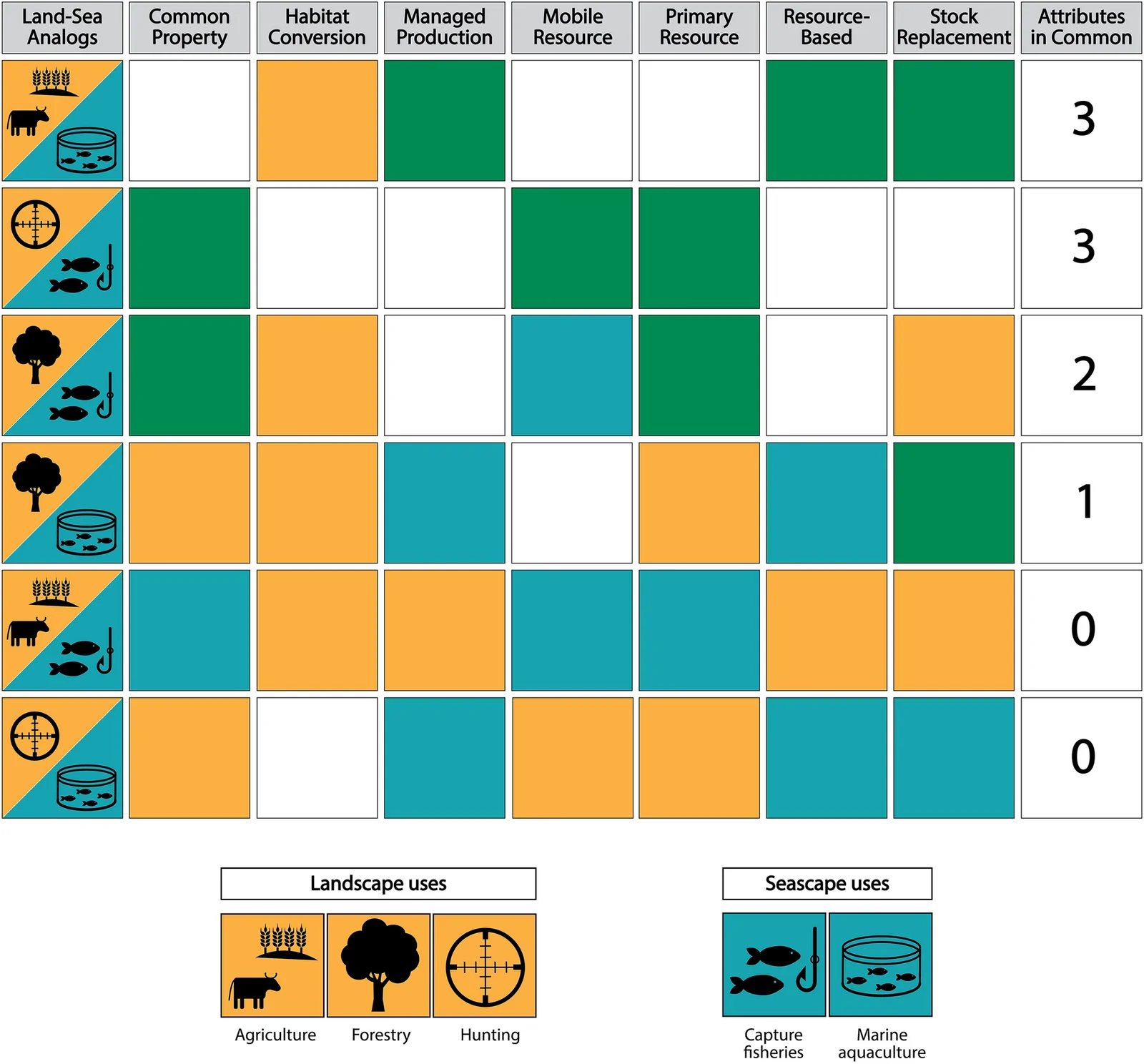
Imperfect land- and seascape analogs. Comparison of resource materialities between land- and seascape uses (e.g., agriculture and fisheries) are shown in the far-left column. Landscape uses and their unique characteristics are indicated in orange, and those characteristics that are specific to seascapes are shown in blue. Characteristics shared by both uses are shown in green. Commonalities across analogs are quantified in the far-right column

Marine aquaculture closely, though imperfectly, resembles the production of food in terrestrial spaces (Fig. 1). Broadly speaking, both marine aquaculture and terrestrial agriculture (inclusive of both crops and livestock) are resource-dependent industries, often requiring inputs such as fertilizer for crops and feed for livestock and fish farming. Further, production across both industries is managed (either extensively or intensively) through mechanisms such as season-based farming cycles and the strategic allocation of inputs (e.g., fertilizer, feed, and pharmaceuticals). After harvest, crops can be replanted, and livestock and marine aquaculture species can be restocked for the next production cycle. A critical point of divergence between marine aquaculture and terrestrial agriculture is that most forms of agriculture (excluding some agroforestry), require habitat conversion as a necessary input to production. The global agriculture system has converted 43% of the earth’s habitable, ice-free land to production (Poore & Nemecek 2018). However, marine aquaculture does not require the wholesale ecological transformation of ocean spaces as a pre-requisite to production in the same way that agriculture does. All types of marine aquaculture production are reliant to varying extents (depending on species and farming method) on the surrounding natural ocean environment to provide fundamental farming inputs. These include, but are not limited to, food for filter-feeding shellfish, nutrients for primary producers like algae, and natural oxygenation for all species (Roels et al. 1979; Tang et al. 2011).

Across the seven resource characteristics, marine aquaculture aligns with forestry through stock replacement mechanisms (i.e., trees can be replanted, and marine aquaculture can be restocked) and has no overlap with hunting.

### Wild-capture fisheries

Wild-capture fisheries and hunting share substantive similarities. Both human uses rely on the extraction of primary resources that are mobile across their respective land- and seascapes. Further, fish and game are fundamentally common property (i.e., non-excludable and rivalrous), though rights-based systems (e.g., individual fish and game quotas or territorial use rights) have been applied, to varying degrees of effectiveness, to prevent overexploitation of these fundamentally open-access resources (Chu 2009; Peeters et al. 2021; Romero & Melo 2021). Commonalities between fisheries and forestry are limited to their extraction of primary resources, and there is no alignment with agriculture.

## Do terrestrial assumptions hold in the sea?

Below, we outline the underlying land-based assumptions of the processes behind intensification and expansion and consider how these assumptions operate in the context of seascape uses.

### Intensification

In land systems, intensification requires key production interventions, such as increasing inputs (e.g., fertilizers or labor), increasing harvest frequency, and/or developing more efficient farming methods (e.g., through technology) to improve productivity (Meyfroidt et al. 2018). In theory, intensification reduces the need to convert more natural habitat to increase production (Clark et al. 2024a, b), thus sparing natural land for conservation (Loconto et al. 2020). In practice, however, empirical evidence supporting the land sparing theory is mixed and, in some cases, contradictory (Rodríguez García et al. 2020). By lowering the marginal costs per unit of production (e.g., operational and labor costs) and consequently product price, intensification can conversely encourage expansion, termed a rebound effect (Meyfroidt et al. 2018). Whether intensification leads to land sparing or expansion depends on factors such as land availability, agricultural policy, and the price elasticity of the intensifying crops (Rodríguez García et al. 2020).

In the context of the land- and seascape uses outlined above, intensification conceptually aligns with 1) resource-based industries that can directly improve productivity by increasing inputs, 2) managed production environments that can increase harvest frequencies and maximize outputs, and/or 3) human uses that can increase crop or stock densities as a means to increase yields. Agriculture strongly maps onto these characteristics, as does marine aquaculture where the intensification of production has the potential to reduce reliance on wild-capture fisheries through the integration of sustainable inputs and harvest practices (Froehlich et al. 2023). The similarities between agriculture and marine aquaculture could suggest potential opportunities for knowledge transfer across land system and sea system science with respect to seafood farming and intensification theory and principles.

However, increasing productivity manifests much differently across human uses that are not amenable to these production interventions (i.e., hunting and fisheries). In the case of wild-capture fisheries, increasing productivity equates simply to killing more fish (Lokrantz et al. 2009; Selgrath et al. 2018). There are no inputs to enhance the productivity of the sea, no controlling the production environment, and no human interventions to systematically replace stocks— there is only fishers’ ability to extract more fish through increased effort and/or technological advancements (Loring 2023). As a common property resource, intensifying fishing activities can decimate targeted populations in the absence of well-designed and implemented institutions with severe consequences for trophic dynamics and ecological functions (Lokrantz et al. 2009). This contradicts the underlying land-sparing assumption that intensification can prevent the expansion of production and protect other natural communities from human use.

### Expansion

While expansion can occur across land that is already subject to human use (e.g., conversion of pastureland to cropland; Cohn et al. 2016), here we focus on the transformation of natural environments for the purposes of production. As highlighted in Sect. “(Imperfect) Land-Sea Analogs” above, agricultural expansion has resulted in the widespread conversion of natural habitats on a global scale. Conversely, though there can be substantial adverse impacts on marine habitats as an *outcome* of production for both marine aquaculture (e.g., Quiñones et al. 2019) and fisheries (e.g., Feitosa et al. 2023), habitat conversion is largely not a necessary *input* to expansion for marine aquaculture or fisheries production in the way it is for agriculture. This is a fundamental distinction regarding the mechanisms of expansion operating across seascape uses.

Expansion in wild-capture fisheries further diverges from the terrestrial context. Fishermen might expand their overall harvest area if they want or need to catch more fish (and intensification is not a feasible alternative) and/or their normal catch volumes are no longer supported by their existing fishing areas, mimicking simplified logics of expansion in agriculture and forestry (Hazell & Wood 2008; Le Polain De Waroux et al. 2018). However, unlike crops, livestock, and forests (but somewhat akin to hunted species), fish are highly mobile and not confined to a particular ‘unit’ of the sea. This dynamic means the location, extent, and tenure of that expansion are exceptionally fluid, and, thereby, more complicated to measure, map, and manage.

## Toward ocean-anchored solution spaces

In our assessment, marine aquaculture aligns most closely with agriculture in that production can be intensified through managed production inputs, controlled production environments, and stock replacement. By contrast, wild-capture fisheries more closely resemble hunting, with production gains primarily achieved from increased extraction effort (e.g., time, labor, and technology) rather than improved per-unit productivity. These commonalities between land- and seascape uses present some scope for knowledge transfer between land system science and a burgeoning sea system science field—though the limited engagement with hunting in the land system science literature means it is largely marine aquaculture that can draw on the terrestrial corpus. Furthermore, these analogs are more strongly overlapping in the processes of intensification than they are in expansion. Expansion in terrestrial systems is defined by land conversion, but the depth and fluidity of the sea and the mobility of marine species create opportunities for human uses and natural environments to more readily coexist. Further, on land, agricultural expansion is shaped by land value, tenure, and market access (as well as biophysical suitability) (Meyfroidt et al. 2018), whereas expansion in the sea is predominantly shaped by the location and movement of valued resources. This difference means that theories regarding the expansion of natural resource use on land are likely unfit for application to seascapes. These substantive divergences across our land-sea analogs underscore that while terrestrial parallels may offer a useful starting point, the development of marine-specific theory is necessary to effectively manage seascapes.

The need for this marine-specific theory is most apparent in the current state of tools for conservation planning and prioritization that are widely applied to seascapes. For example, systematic conservation planning is a framework for making decisions about the type and location of management interventions, informs a suite of terrestrial conservation tools, and has been widely applied to marine contexts, including the design of the Great Barrier Reef Marine Park and marine spatial planning initiatives in the Baltic Sea and off the coast of southwest Africa (Finke et al. 2020; McIntosh et al. 2017). Systematic conservation planning has three core underlying principles (land sparing, cost optimization, and fixed spatial tradeoffs) that are at least partially incongruous with marine contexts (Margules & Pressey 2000; Watson et al. 2011). Land sparing is predicated on intensification in one location allowing for the conservation of another. As discussed above, the mechanisms of intensification that underpin land sparing do not manifest in fisheries, though they may be observed in marine aquaculture. Cost optimization assumes value is attributed to specific parcels; however, value is generally attributed to marine resources themselves, not the areas in which they are produced. And finally, while fixed parcels can be defined in seascapes, the social-ecological fluidity of the sea compromises their management application. Moreover, fixed spatial tradeoffs assume the presence of clear jurisdictional authority, which in marine contexts is often split among national agencies, regional fisheries management organizations, and other ocean users with different objectives, data standards, and enforcement capacities.

We also consider how concepts of leakage and spillovers manifest differently between land- and seascapes. Leakage refers to the displacement of extractive activities from regulated or protected areas to other locations (thereby eroding conservation gains at larger scales) and is a well-established concern in both theoretical and empirical work on land-use and terrestrial management (Guizar‐Coutiño et al. 2022; Meyfroidt et al. 2018, 2020). In landscapes, systematic conservation planning views these outcomes as a negative externality and seeks to mediate leakages through the strategic prioritization of fixed land parcels characterized by relatively immobile production systems and well-defined property rights. In marine systems, leakage similarly manifests as the redistribution of human uses (most commonly fishing effort) away from managed spaces and into surrounding or distant seascapes (Cunningham et al. 2016). However, given mobile resources, fluid boundaries, and overlapping jurisdictions, marine leakage is often more diffuse and more difficult to untangle from ecological responses to protection. For example, while marine protected areas might displace fishing efforts to waters adjacent to or more distant from the designated conservation space (e.g., Rees et al. 2021; Stevenson et al. 2013), there is also evidence of positive effects where marine protected areas reduce fishing effort in surrounding areas (e.g., McDonald et al. 2024). The determinants of this redistribution (or lack thereof) vary broadly, from the relative mobility of fish and fishers to fishery recovery and spillover rates (Cabral et al. 2017; Costello 2024; Kellner et al. 2007). Thus, while leakage in both land- and seascapes can reflect a common process of displacement of human use, terrestrial planning frameworks tend to assume leakage is exogenous and preventable, whereas marine systems reveal it to be an intrinsic dimension of managing mobile resources in open systems.

Systematic conservation planning is just one applied example of theoretical mismatch in the deployment of terrestrial concepts in marine spaces, but it is emblematic of how ubiquitously land-based theory informs decision making in seascapes. A sea system science can inform our interrogation of these land-based management concepts and tools by engaging with the distinct socioecological assemblages created in and by seascapes and explaining the connections and relationships between people and the sea. In doing so, seascape theory can inform an alternative marine management design ontology: one that is mobility aware, temporally adaptive, and nimble across seascape uses and jurisdictions. This seascape management ontology should not be anti-spatial but rather emphasize process-oriented solutions that target functional ecological resilience and social equity (Anthony et al. 2015; Blythe et al. 2026) while being space-aware. Emerging agile spatial management measures, such as dynamic ocean management, moving marine protected areas, and real-time bycatch avoidance, should be coupled with seascape use rights and participatory action to flexibly and equitably link ecological outcomes to people and communities.

Seascape ecology is a critical mechanism for actioning this new, sea system science-informed management ontology—providing the tools and methodologies to translate conceptual findings into applied outcomes. By explicitly treating marine spaces as socially and ecologically fluid systems rather than fixed places, seascape ecology links pattern-process relationships across both space and time (e.g., habitat connectivity, productivity gradients, patch size, edge length, corridor structure) with human dimensions of seascape use (e.g., fishing effort, farming density) and climatic variability (Hidalgo et al. 2016). As a sea system science-informed management approach emphasizes accurately and nimbly representing coupled social-ecological dynamics, seascape ecology can enable the deployment of parametric rather than strictly numerical and spatially explicit management paradigms (Acheson & Wilson 1996). For example, fishing pressure can be modulated relative to habitat connectivity, protection can be dynamically scaled in response to climate stressors, and areas with high regenerative capacity can be prioritized for targeted management responses. By identifying sensitive spatial levers, feedbacks, and thresholds, seascape ecology operationalizes sea system science while also creating opportunities to test and refine its theoretical concepts through real-world applications.

Seascape ecology is further positioned to drive co-evolution between empirical observation and theory development through large, integrated datasets that are increasingly available in marine systems (though largely missing from terrestrial contexts). Global vessel tracking platforms and the widespread adoption of vessel monitoring systems has enabled detailed reconstructions of fishing activity through reported positions or inferred vessel tracks (Lee et al. 2010; Witt & Godley 2007), improving our understanding of who is fishing where and informing more effective governance (Orofino et al. 2023). When combined with broad-scale oceanographic datasets (e.g., temperature, currents, and nutrient flows), these data allow fishing activity and other seascape uses to be modelled in relation to dynamic environmental conditions. This integration enables spatially and temporally adaptive management responses to shifting species distributions, mobile seascape uses, and moving habitats (Lewison et al. 2015; Maxwell et al. 2015). Critically, as seascape ecology operationalizes these theory-informed management interventions, it produces empirical evidence that can further refine sea system science concepts and dynamic management approaches. In this way, seascape ecology and sea system science can co-evolve through iterative cycles of intervention, observation, and adaptation.

## Conclusion

Seascape ecology has the requisite methodologies and tools for understanding the distinct patterns and processes that operate across marine systems. However, engaging with how humans use, govern, and transform marine spaces has, to date, remained beyond the discipline’s purview. A co-evolving sea system science would address these gaps and generate the necessary knowledge for managing seascapes as complex, fluid social-ecological systems. Without this integration, seascape ecology risks reinforcing management solutions derived from terrestrial theory that are procedurally feasible in seascapes but materially misaligned. With it, the two fields can inform an alternative marine management ontology that is responsive to fluid boundaries, mobile resources, shifting access, and overlapping jurisdictions.

Advancing sea system science as a distinct research arena will require further and more in-depth theoretical and empirical exploration of the land-sea tensions proposed here. However, the broader management impacts of a sea system science corpus will necessitate institutional recognition of the distinctiveness of seascapes by the entities managing marine spaces with the existing suite of terrestrially-derived tools and strategies. Seascape ecology, as an established discipline that already informs marine management approaches, can serve as a conduit for integrating sea system science ontologies into existing governance frameworks.

The value of sea system science extends beyond advancing marine management design. Seascapes expose assumptions of social-ecological systems and sustainability theory that are derived from landscape contexts. As such, oceans function not only as sites of evaluation for landscape-derived frameworks but also as large-scale testbeds for social-ecological theory, with opportunities to generate transferable insights related to terrestrial sustainability challenges. For example, the close approximation of wild-capture fisheries and hunting in our land-sea analogs, and the relative absence of the latter in the existing land system science discourse, presents an opportunity for sea system science to inform new modes of thinking about land use dynamics related to mobile, common property resources. In this way, sea system science positions seascapes not as an extension of or an exception to landscapes but as their own distinct human–environment systems that both necessitate new theoretical considerations and challenge fundamental assumptions of social-ecological change.

## Data Availability

No datasets were generated or analysed during the current study.
